# Role of Fasting in the Management of Non-alcoholic Fatty Liver Disease (NAFLD): A Systematic Review of Clinical Trials

**DOI:** 10.7759/cureus.84259

**Published:** 2025-05-16

**Authors:** Ryan R Haddad, Naga Spandana Battula, Timmie Chay, Tirath Patel, Nabina Dumaru, Srivarshini Maddukuri, Safeera Khan

**Affiliations:** 1 Clinical Research, California Institute of Behavioral Neurosciences and Psychology, Fairfield, USA; 2 College of Medicine, American University of Antigua, St. John, ATG; 3 Radiology, Frimley Park Hospital, Camberley, GBR; 4 Internal Medicine, Dr. D.Y. Patil Medical College, Hospital and Research Centre, Pune, IND; 5 Family Medicine, Michener Institute at University Health Network (UHN), Toronto, CAN

**Keywords:** “intermittent fasting”, liver steatosis, nonalcoholic fatty liver disease (nafld), religious fasting, time restricted intermittent fasting

## Abstract

Non-alcoholic fatty liver disease (NAFLD) is one of the leading causes of chronic liver disease. Lifestyle changes, especially dietary, have been recognized as important measures for managing the condition. This systematic review aimed at assessing the effectiveness of fasting protocols, including time-restricted feeding (TRF), alternate-day fasting (ADF), the 5:2 diet, and other lifestyle interventions in enhancing metabolic and hepatic profiles. The search for randomized controlled trials (RCTs) was extended to include PubMed, Scopus, ScienceDirect, and Google Scholar for articles published between 2019 and 2024. The studies that included participants with fatty liver disease and examined the effects of fasting on hepatic steatosis, metabolic markers, and liver enzymes were included, and the quality of the studies was evaluated using the Cochrane risk of bias tool. Out of the 12 RCTs that were included in the study, there was a significant reduction in hepatic steatosis, an increase in insulin sensitivity, and a decrease in inflammatory markers. TRF and ADF were found to be effective forms of calorie restriction, as they were well tolerated by the patients. The combination of ADF with aerobic exercise and TRF with low-sugar diets was seen to have potent effects on the liver and other metabolic parameters. However, certain drawbacks include the variation in the fasting protocols, small subject numbers, and brief follow-up periods. The current study highlights that intermittent fasting is a viable non-pharmacological option for the management of NAFLD with a focus on the timing and content of eating periods. The following are needed to strengthen the evidence and to guide the clinicians in its application: standardized fasting protocols and long-term trials.

## Introduction and background

Non-alcoholic fatty liver disease (NAFLD) is the leading cause of chronic liver disease (CLD) [1]. NAFLD affects 25% of the global adult population [2]. The rise in NAFLD incidence is closely linked with the increase in metabolic disorders such as obesity, type 2 diabetes mellitus (DM), and insulin resistance, hence becoming a major public health concern [3]. As NAFLD has no specific pharmacological therapy management, the primary recommended treatment is lifestyle changes, especially weight loss through diet and exercise [4].

Intermittent fasting (IF) has become popular as a dietary approach for the management of NAFLD, as it may have more favorable cardiometabolic effects than conventional calorie restriction [4]. IF is a form of dietary intervention that involves various protocols, including time-restricted feeding (TRF), alternate-day fasting (ADF), and modified alternate-day calorie restriction (MACR), where individuals alternate between periods of eating and fasting. The above strategies have been shown to work in the reduction of body weight, improvement of liver enzyme levels, and reduction of hepatic steatosis [5].

Among these therapies, one of the most effective is TRF, which is a form of calorie restriction where an individual confines their food intake to specific time windows each day and has been found to enhance liver function as well as control other metabolic disorders in patients with NAFLD. The TRF diet plan, along with a low sugar diet, resulted in a significant reduction of liver fat, together with the improvement of lipid and inflammatory markers when compared with the control group [6]. The same results have been observed with other forms of fasting, including religious fasting; for instance, the Ramadan fasting, which is IF from dawn to sunset. Studies have supported that Ramadan fasting positively affected the hepatic transaminases and lipid profiles of patients with NAFLD [3].

The following are the potential therapeutic approaches that have been investigated for the management of NAFLD: One of the variants of IF is MACR, and its effectiveness in the management of NAFLD has been evaluated [5]. An eight-week randomized controlled trial (RCT) on the MACR diet showed improved liver steatosis, BMI, and liver function tests compared to the control group that followed a regular diet [5].

The findings indicate that IF regimes, such as MACR, may provide a useful alternative for patients who have difficulty adhering to conventional calorie-restricted diets. The timing of energy consumption is increasingly recognized as a significant element in managing metabolic disorders, including NAFLD [7]. The recommendation to align feeding patterns to a circadian rhythm stems from the understanding that energy metabolism and other physiological processes are influenced by circadian rhythm [8]. One of the most popular dietary strategies that is based on the circadian rhythm is time-restricted eating (TRE). This strategy has been shown to be effective in better glycemic control and lipid profile, as well as reducing hepatic steatosis in patients with NAFLD [2]. In conclusion, lifestyle changes, especially those incorporating IF and alterations in food intake timing, have shown potential in the management of NAFLD. This comprehensive review seeks to evaluate the efficacy of different IF regimens, including TRF, MACR, and Ramadan fasting, in enhancing liver function and metabolic health in persons with NAFLD. The findings from this research may aid in the development of personalized and sustainable dietary approaches for managing NAFLD.

## Review

Methods

This systematic review was conducted in accordance with the Preferred Reporting Items for Systematic Reviews and Meta-Analyses (PRISMA) 2020 guideline for systematic review and meta-analysis [9]. The databases used are PubMed, Google Scholar, Scopus, and ScienceDirect. The final search was conducted on the 25th of October, 2024. Only studies published in English between 2019 and 2024 were included. The combination of keywords and Medical Subject Headings (MeSH) used in PubMed and other databases is summarized in Table 1:

**Table 1:** Number of articles identified from individual databases MeSH: Medical Subject Headings

Search strategy	Database used	Number of papers identified
Fasting OR Intermittent Fasting OR Calorie restriction OR Protein restriction OR Water fasting AND Non-Alcoholic fatty liver disease OR NAFLD OR steatosis OR Metabolic associated fatty liver disease OR MAFLD	Scopus	1390
Fasting OR Intermittent Fasting OR Calorie restriction OR Protein restriction OR Water fasting AND Non-Alcoholic fatty liver disease OR NAFLD OR steatosis OR Metabolic associated fatty liver disease OR MAFLD	Science Direct	22,304
Fasting OR Intermittent Fasting OR Calorie restriction OR Protein restriction OR Water fasting AND Non-Alcoholic fatty liver disease OR NAFLD OR steatosis OR Metabolic associated fatty liver disease OR MAFLD	Google Scholar	18,200
Fasting OR Intermittent Fasting OR Calorie restriction OR Protein restriction OR Water fasting AND Non-Alcoholic fatty liver disease OR NAFLD OR steatosis OR Metabolic associated fatty liver disease OR MAFLD	PubMed	772
("Fasting/adverse effects"[Majr] OR "Fasting/metabolism"[Majr] OR "Fasting/physiology"[Majr]) AND ("Non-alcoholic Fatty Liver Disease/diet therapy"[Majr] OR "Non-alcoholic Fatty Liver Disease/drug therapy"[Majr] OR "Non-alcoholic Fatty Liver Disease/prevention and control"[Majr] OR "Non-alcoholic Fatty Liver Disease/therapy"[Majr])	PubMed MeSH	2

Eligibility and Selection of Studies 

A comprehensive screening of titles and abstracts was carried out. To be included in this study, articles had to meet the following criteria: they must be full-text, freely accessible, written in English, and published between 2019 and the present. Only RCTs that aimed at understanding the link between fasting and NAFLD were included, and the studies could involve human participants of any age, ethnic background, or region. Excluded from the review were editorials, conference posters, animal studies, and gray literature. Any study meeting the inclusion criteria was deemed suitable for review.

Data Extraction and Quality Assessment

The search results were exported to Rayyan, where two independent reviewers screened the titles and abstracts. Full-text articles were retrieved and assessed for eligibility. The selection process was documented using the PRISMA flow diagram. Full texts of the final article were collected for quality assessment and data extraction. The data extraction aimed at capturing key information of the studies, including the design of the study, the number of participants, the baseline characteristics, the type of fasting and the duration of treatment, the average follow-up time for each participant group, the outcomes of the study, and the source of funding, which could be pharmaceutical or otherwise. The Cochrane risk of bias tool was used to assess the quality of the studies, where only those with no high risk of bias in all the domains were considered for the review. Any differences during this process were debated and agreed upon to reach a consensus.

Results

**Figure 1:**
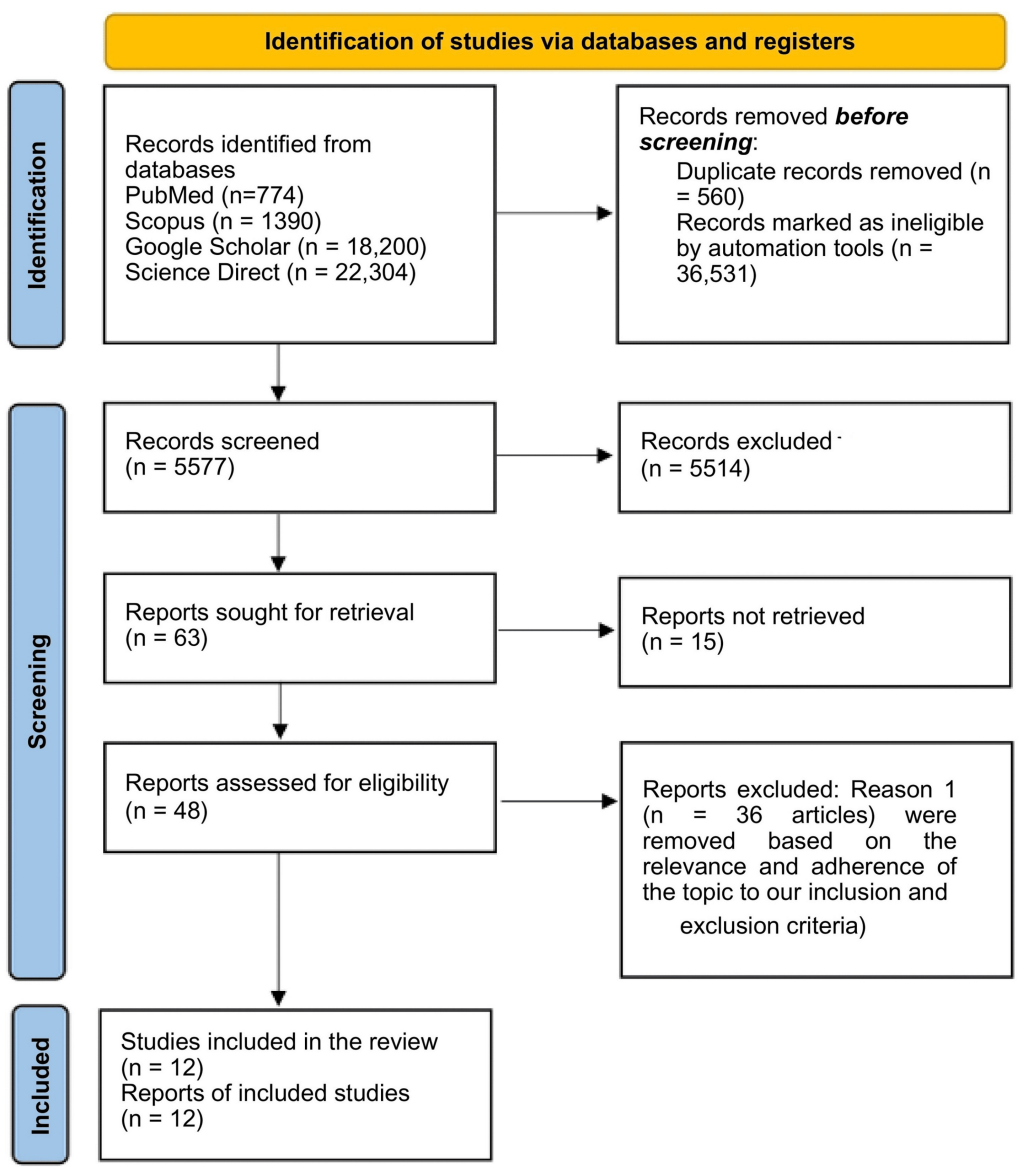
Preferred Reporting Items for Systematic Reviews and Meta-Analyses 2020

After doing a database search, we got a total of 42,668 articles. We eliminated 36,531 using automation tools. Then we identified 560 duplicates, filtered the titles of 5577 articles, and excluded 5514. This led to the selection of 63 research papers. Of the 36 articles reviewed in detail and excluded from the study based on their relevance and adherence to the research topic, we were left with 12, as shown in Figure 1. The 12 articles have been compiled in Table 2 with the key characteristics and conclusions.

**Table 2:** Characteristics of studies included in this systematic review NAFLD: non-alcoholic fatty liver disease; ALT: alanine aminotransferase; AST: aspartate aminotransferase

Author	Journal and publication year	Purpose of study	Total participants	Conclusion	Type of research
Goss et al. [10]	Pediatric Obesity, 2020	To determine if a carbohydrate-restricted diet can reduce liver fat and enhance metabolic health more than a conventional fat-restricted diet. ​	32	The results show that a moderately low-carbohydrate diet may help to decrease the amount of fat in the liver, increase lean body mass, and improve insulin sensitivity in children with non-alcoholic fatty liver disease without calorie restriction.	A pilot randomized trial
Liu et al. [11]	Nutrition Journal, 2024	To assess the impact of intensive lifestyle modification on liver steatosis as determined by alterations in the fat attenuation parameter, as well as other metabolic parameters.	226	This randomized controlled trial (RCT) showed that a 12-week intensive lifestyle intervention program can reduce liver steatosis and other metabolic disorders in overweight and obese Chinese patients with nonalcoholic fatty liver disease.	Multicenter randomized controlled trial in China
Chen et al. [12]	Asia Pacific Journal of Clinical Nutrition, 2020	The purpose of the study was to assess the effect of a high-fiber and low-carbohydrate diet as well as nutritional counseling on body composition, liver function tests, metabolic parameters, and the severity of hepatic steatosis in patients with non-alcoholic fatty liver disease.	44	A low-carbohydrate, high-fiber diet along with educational intervention is useful in the management of non alcoholic fatty liver disease as it leads to a reduction in weight and fat as well as metabolic markers, including liver enzymes, blood glucose, blood lipids, and uric acid. Women showed a better outcome in these parameters than men.	Randomized controlled trial
Cohen et al. [13]	Journal of Clinical Investigation, 2021	To determine whether a decrease in sugar intake can decrease hepatic de novo lipogenesis and the related metabolic disorders in adolescents with NAFLD.	29	The results show that a sugar-restricted diet reduces hepatic lipogenesis, fasting insulin levels, hepatic fat, and ALT in adolescents with non-alcoholic fatty liver disease (NAFLD).	Randomized controlled trial
Feehan et al. [14]	Nutrients, 2023	What are the effects of time-restricted fasting compared to the standard treatment on hepatic steatosis, visceral adiposity, and other aspects of the metabolic profile?	32	Time-restricted fasting offers superior improvements in patients with NAFLD, improving steatosis, weight, and waist circumference despite a lack of change in overall caloric intake.	A single-blinded crossover trial
Ezpeleta et al. [15]	Cell Metabolism, 2023	Is the combined effect of alternate-day fasting and exercise more effective in the reduction of hepatic fat and other metabolic risk factors than either of the two alone?	80	The results of this randomized controlled trial demonstrate that alternate day fasting combined with aerobic exercise is an effective lifestyle therapy to reduce intrahepatic triglyceride content versus exercise alone and controls.	Randomized controlled trial
Wie et al. [16]	JAMA Network Open, 2023	The aim was to understand if a time-restricted eating pattern provided an added advantage over the standard calorie restriction for the management of liver fat and other aspects of metabolic disorder in patients with NAFLD.	88	Among adults with obesity and NAFLD, time-restricted eating did not produce additional benefits for reducing intrahepatic triglyceride content, body fat, and metabolic risk factors compared with daily calorie restriction.	Randomized clinical trial
Johari et al. [5]	Scientific Reports, 2019	To assess whether modified alternate-day calorie restriction has an impact on BMI, liver enzymes, and liver steatosis.	43	Eight weeks of modified alternate-day calorie restriction strategy appears more effective than a usual habitual diet in the control of NAFLD activity and with a good adherence rate.	Randomized controlled trial
Cai et al. [17]	BMC Gastroenterology, 2019	To determine whether the two interventions, the alternate day fasting and the time-restricted feeding, are safe and effective as dietary interventions for the treatment of NAFLD and other related metabolic syndromes.	271	Alternate-day fasting appears to be an effective diet therapy for individuals with NAFLD that can achieve weight loss and improvement of dyslipidemia within a relatively short period (four to 12 weeks).	Randomized controlled trial
Holmer et al. [18]	JHEP Reports, 2021	To assess the effect of the 5:2 and a low-carbohydrate, high-fat diet on hepatic steatosis as assessed by magnetic resonance spectroscopy, as well as other secondary outcomes such as liver stiffness, insulin resistance, and blood lipids.	74	The low-carb high-fat diet and the 5:2 diet produced better outcomes on steatosis and body weight than the standard lifestyle advice provided by a hepatologist to patients with NAFLD. These findings suggest that dietary recommendations can be provided according to individuals’ choices to get better outcomes.	Randomized controlled trial
Hekmatdoost et al. [19]	Frontiers in Nutrition, 2022	To evaluate the impacts of the 5:2 intermittent fasting on liver steatosis and fibrosis, body composition, lipid profile, and the inflammatory biomarkers.	44	Adhering to the 5:2 intermittent fasting diet has been shown to result in weight loss as well as positive changes in anthropometric measures of obesity. It also has the benefit of improving liver enzymes such as ALT, AST, bilirubin, and reducing liver fat, triglycerides, and levels of inflammatory cytokines in patients suffering from non alcoholic fatty liver disease.	Randomized controlled trial
Kord-Varkaneh et al. [6]	Nutrition, 2023	To evaluate the impact of time-restricted feeding and the low-sugar diet on body composition, liver function tests, cardiometabolic and inflammatory markers.	52	Time-restricted feeding plus a low-sugar diet can reduce adiposity and improve liver, lipid, and inﬂammatory markers in patients with NAFLD.	Randomized controlled trial

**Figure 2:**
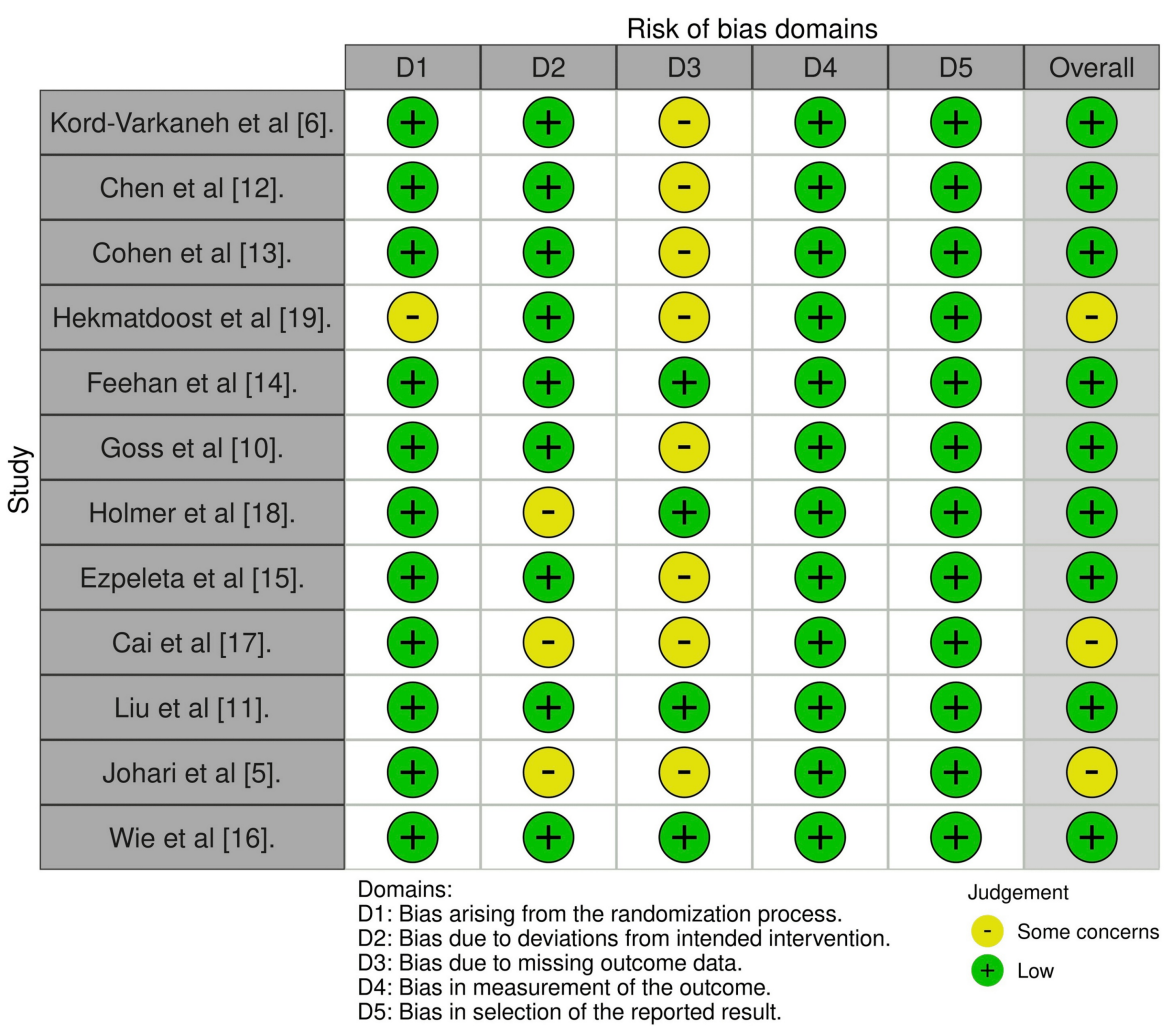
Risk-of-bias 2 Cochrane collaboration's tool for risk-of-bias assessment of randomized controlled trials [5,6,10,11,12,13,14,15,16,17,18,19]

Following a thorough examination of all articles, we utilized the risk-of-bias 2 Cochrane quality assessment tool to evaluate the remaining 12 papers [20]. This tool assessed multiple domains of potential bias in RCTs and categorized the studies as having low, some concerns, or high risk of bias. Each included RCT was evaluated based on five key domains: bias arising from the randomization process, bias due to deviations from intended interventions, bias due to missing outcome data, bias in outcome measurement, and bias in the selection of reported results. The evaluation results of these articles are presented in Figure 2.

Discussion

The twelve RCTs included in this review evaluated various interventions for managing NAFLD. Four trials focused on low-carbohydrate or low-sugar diets [6,10,12,13], four trials focused on different IF protocols [5,14,17,19], two trials focused on combined interventions [15,18], and the last two trials assessed the effects of broad lifestyle interventions [11,16].

Low-Carbohydrate or Low-Sugar Diets

Dietary modifications and TRF are effective measures for the management of NAFLD. For example, Chen et al. [12] found that a low-carbohydrate, high-fiber diet was effective in enhancing the metabolic profile, such as insulin resistance, liver enzymes, and body fat. They also found that there were some gender differences in the results, where certain biomarkers improved more in females than males. This highlights the need to take into account demographic characteristics such as gender when creating dietary interventions. In a similar manner, [13] showed that an eight-week sugar-restricted diet can decrease hepatic de novo lipogenesis and hepatic fat in adolescents with non-alcoholic fatty liver disease. This study not only supported the findings of previous research, which pointed to sugar as a major factor in the development of NAFLD, but also established a statistical link between a decrease in sugar consumption and improvement in insulin and alanine transaminase levels. The results thus support the notion that dietary interventions that aim at reducing intake of sugars, especially the simple carbohydrates, should be targeted at younger individuals as well. More evidence by Goss et al. [10] also confirmed the effectiveness of carbohydrate-restricted diet (CRD) in adolescents, as they also found that CRD led to a decrease in hepatic lipids and subcutaneous abdominal fat and improved insulin resistance without the restriction of calorie intake. This implies that the kind of macronutrients that one takes is more important than the amount of calories taken [6]. 

Further enhanced these conclusions by revealing that the concomitant use of TRF and low-sugar dieting had superior beneficial effects. There was a significant reduction in body fat, high-sensitivity C-reactive protein (hs-CRP), hepatic steatosis, and cytokeratin-18, and an additional improvement in the liver enzyme profiles and the cardiometabolic biomarkers. Thus, the outcomes of Wei et al. [16] highlight the possible symbiotic effect of the dietary composition and feeding schedule in optimizing the metabolic and hepatic health. Thus, it indicates that there is a need to develop NAFLD management strategies that take into consideration the quality of the diet as well as the schedule of the meals and that are also influenced by demographic and metabolic characteristics of the individual.

Various Intermittent Fasting Protocols

Of the twelve clinical trials in this review, four investigated the effects of different forms of IF, TRF, ADF, MACR, and the 5:2 diet on NAFLD. Cai et al. [17] showed that ADF resulted in a weight loss and brought about improvements in the lipid profile with a reduction in triglycerides and LDL cholesterol within 12 weeks. Consistent with that, Feehan et al. [14] discovered that TRF led to a reduction in hepatic steatosis and waist circumference without the need for calorie restriction when compared to standard care. Therefore, both studies indicate that IF has the potential to reverse the effects of NAFLD through weight loss and improved metabolic profiles. Hekmatdoost et al. [19] found that the 5:2 fasting reduced hepatic steatosis, liver enzymes, and triglycerides as well as improved the levels of inflammatory markers after 12 weeks. Johari et al. [5] found a reduction in BMI, liver fibrosis, and steatosis scores in eight weeks, showing that IF is feasible and an effective dietary intervention. All these findings highlight the fact that the IF diets are viable and convenient options compared to continuous caloric restriction. It is also noted that IF options can enhance the patients’ compliance and provide positive outcomes in the management of NAFLD.

These studies demonstrate that IF can target major pathways involved in the development of NAFLD, such as insulin resistance, visceral fat, systemic inflammation, and cytokines. Although variations in fasting protocols and research methods make a straight comparison impossible, the positive changes in liver condition and metabolic parameters support the notion that IF is an effective non-drug intervention for NAFLD management.

Combined Interventions

The effects of ADF combined with moderate-intensity aerobic exercise for three months were effective in decreasing intrahepatic triglyceride by 5.5%, increasing insulin sensitivity, losing weight, and reducing ALT, thus suggesting that the combination might be more effective than exercise alone [15]. Similarly, Holmer et al. [18] discovered that the 5:2 fasting diet plan was as efficient as the low-carb, high-fat (LCHF) diet in reducing liver fat and weight and improving liver stiffness and LDL cholesterol levels compared to standard care. Additionally, patients on the 5:2 diet have been shown to have better adherence.

Intensive lifestyle intervention (ILI) that included a low-carbohydrate, high-protein, calorie-restricted diet, exercise, and personalized counseling produced significant changes in fat attenuation parameter (FAP) and BMI after 12 weeks when compared to a balanced calorie-restricted diet [11]. This holistic approach also had positive effects on metabolic markers, including alanine aminotransferase (ALT), aspartate aminotransferase (AST), and fasting blood glucose, and therefore has potential for managing NAFLD in different populations. On the other hand, Wei et al. [16] directly compared TRE with daily calorie restriction and revealed that there were no significant differences in the intrahepatic triglyceride score or metabolic improvements after one year of intervention, therefore underlining the importance of caloric restriction regardless of the meal timing schedule.

The review has some concerns; the inconsistency in the fasting protocols, small subject numbers, and short follow-up periods in some of the studies are major concerns as they reduce the external validity of the results.

## Conclusions

This systematic review explored the use of IF protocols, including TRF, ADF, and the 5:2 diet, and other dietary and lifestyle interventions in the management of NAFLD. These interventions have been demonstrated to significantly positively affect the major metabolic parameters, decrease hepatic steatosis, and increase the overall liver function. The two plans that include TRF and ADF appear particularly promising in addressing key pathological mechanisms of NAFLD, including insulin resistance, central adiposity, and systemic inflammation, which are the pathological processes that lead to NAFLD development, and thus may be considered potential alternatives to calorie restriction. The evidence also suggests that the composition of the diet may be as important as the timing of food intake. Combined strategies, such as TRF with reduced sugar intake, showed greater efficacy than either approach in isolation. In addition, ILI and combined approaches, for instance, ADF combined with aerobic exercise, produced positive and strong effects on metabolism and liver disease, highlighting the benefits of the tailored multifaceted approaches.

Future research should focus on the standardized IF protocols, the long-term effects, and the demographic factors like age, gender, and cultural dietary practices. In summary, IF represents a promising non-pharmacological strategy for managing NAFLD. While current findings are encouraging, long-term and large-scale trials are needed to confirm its role as an effective and sustainable treatment. Such research could pave the way for developing accessible, cost-effective, and culturally adaptable dietary interventions to support the global management of NAFLD. 
